# The slope of assimilation rate against stomatal conductance should not be used as a measure of water use efficiency or stomatal control over assimilation

**DOI:** 10.1007/s11120-023-01054-6

**Published:** 2023-10-30

**Authors:** Chandra Bellasio

**Affiliations:** 1 Biology of Plants Under Mediterranean Conditions, Department of Biology, University of the Balearic Islands, 07122 Palma, Illes Balears Spain; 2 Laboratory of Theoretical and Applied Crop Ecophysiology, School of Biology & Environmental Science, University College Dublin, Belfield, Dublin 4, Ireland; 3 Research School of Biology, The Australian National University, Acton, ACT 2601 Australia

**Keywords:** Photosynthesis, Analysis, Response, Limitation, Optimality

## Abstract

Quantifying water use efficiency, and the impact of stomata on CO_2_ uptake are pivotal in physiology and efforts to improve crop yields. Although tempting, relying on regression slopes from assimilation-stomatal conductance plots to estimate water use efficiency or stomatal control over assimilation is erroneous. Through numerical simulations, I substantiate this assertion. I propose the term ‘instantaneous transpiration efficiency’ for the assimilation-to-transpiration ratio to avoid confusion with ‘intrinsic water use efficiency’ which refers to the assimilation-to-stomatal conductance ratio, and recommend to compute both metrics for each gas exchange data point.

Stomata regulate the rate of water vapour escaping leaves (transpiration, *E*) by adjusting stomatal aperture, inevitably restricting the rate of CO_2_ uptake, called assimilation (*A*). This results in a difference in CO_2_ concentration from higher atmospheric (*C*_a_), to lower internal concentration (*C*_i_). The ease with which water vapour permeates stomata is referred to as stomatal conductance to water (*g*_SW_), which is higher than that for CO_2_ due to the smaller size of the H_2_O molecule (*g*_SW_≈1.6*g*_SC_). The theoretical basis for identifying the extent to which stomata restrict assimilation was laid by Gaastra (1959) and further developed by Jones (1985). Stomatal restriction was associated to sensitivity: how much *A* varies in response to a change in *g*_SC_. Mathematically, the latter is achieved by expressing *A* as a function of multiple variables and calculating the change in *A* resulting from a small alteration of *g*_SC_, while keeping all other variables unchanged. In formal notation, this is the partial derivative, $\partial A/\partial {g}_{\mathrm{SC}}$.

The trade-off between assimilation and the rate of water loss can be represented by a similar metric, the marginal carbon revenue of water, $\partial \mathrm{A }/ \partial E$ (Buckley et al. 2017), or, alternatively, by water use efficiency. The notion of water use efficiency had its origins in agronomy, and measured the production (grain harvested, above ground biomass, total plant or biomass) per unit of water used (water supplied through irrigation, rain, water transpired, or evapotranspiration) (Hatfield and Dold 2019). At the leaf level, two formulations of water use efficiency are commonly used. The ratio between assimilation and *g*_SW_ is referred to as intrinsic water use efficiency (_i_WUE), while the ratio between assimilation and transpiration is often termed instantaneous water use efficiency. However, this terminology can lead to confusion. To mitigate this ambiguity, I encourage using the term ‘instantaneous transpiration efficiency’ (ITE) for the ratio between *A* and *E*, as suggested by Duursma et al. (2013). While _i_WUE is dependent solely on leaf properties, ITE increases without bound as air humidity rises, irrespective of the plant. This is because transpiration is the product of* g*_SW_ and the air-to-leaf water mole fraction gradient, *D*_S_ $( {\text{ITE}}= {}_{\mathrm{i}}{\text{WUE}}/{D}_{\mathrm{S}})$. Consequently, _i_WUE is typically favoured over ITE when comparing plants (Table 1).

**Table 1:** Acronyms, definitions, variables, and units used

Symbol	Definition	Units
*A, A*_op_	Net assimilation, unspecified or measured under ordinary, operational conditions	μmol CO_2_ m^−2^ s^−1^
*E*	Leaf level water transpiration rate $E ={g}_{\mathrm{S}} {D}_{\mathrm{S}}$	mmol H_2_O m^−2^ s^−1^
*A*_SAT_*, A*_SAT_ʹ	CO_2_-saturated *A* in absence or in presence of *L*_NS_, respectively,	μmol CO_2 _m^−2^ s^−1^
*C*_a_	CO_2_ concentration outside the leaf	μmol CO_2_ mol air^−1^
CE, CEʹ	Initial slope of the *A*/*C*_i_ curve in absence or in presence of *L*_NS_, respectively	mol air m^−2^ s^−1^
*C*_i_*, C*_iop_	CO_2_ concentration in the substomatal cavity as calculated by the infra-red gas exchange analyser, unspecified, or under ordinary operational conditions	μmol CO_2_ mol air^−1^
_i_WUE	Intrinsic water use efficiency ${}_{\mathrm{i}}{\text{WUE}}=A/{g}_{\mathrm{S}}$	μmol CO_2_ mol air ^−1^
ITE	Instantaneous transpiration efficiency;${\text{ITE}}=A/E = {}_{\mathrm{i}}{\text{WUE}}/{D}_{\mathrm{S}}$	μmol CO_2_ mol H_2_O^−1^
*D*_S_	Water vapour mole fraction difference between the leaf and air	mmol H_2_O mol air^−1^
*g*_S_*, g*_SC_* g*_SW_	Stomatal conductance, in general, to CO_2_, or to water vapour, respectively	mol air m^−2^ s^−1^
*L*_S_	Stomatal limitation to photosynthesis	Dimensionless
*L*_NS_	Stomatal limitation to photosynthesis	Dimensionless
*Γ*	*C*_i_‒A compensation point, *i.e., C*_i_ at which *A* = 0	μmol CO_2_ mol air ^−1^
*ω*	Curvature of the non-rectangular hyperbola describing the* C*_i_ dependence of *A*	Dimensionless

Regression analysis, a widely employed statistical technique, offers a easily accessible approach for exploring relationships between variables. The output of regression analysis is the slope and the intercept of the line that best fits this relationship, and, optionally, the associated statistics. Unfortunately, confusion between the partial derivative, the slope of the regression, and the average value of the datapoints used for the regression has led to the incorrect utilization of regression analysis as a substitute for sensitivity analysis or water use efficiency. I will present two instances of these concepts being confounded.

## The slope of the regression of *A* on *g*_SC_ does not measure stomatal control over assimilation

The simple argument that if plants with a steeper slope in the regression of *A* against *g*_SC_ were to increase *g*_SC_, they would enhance *A* more than plants with a lower slope has been frequently put forth (Yan et al. 2016; Kawamitsu et al. 1993; Carriquí et al. 2015), but is actually incorrect. Assimilation typically responds to multiple factors, therefore $\partial A/\partial {g}_{\mathrm{SC}}$ captures the incremental change in CO_2_ assimilation resulting from a slight alteration in stomatal conductance to CO_2_, while maintaining other factors constant. However, in experimental conditions, any change observed in *g*_SC_ is typically not independent of other variables affecting *A*. Instead, *g*_SC_ may respond to environmental drivers that typically also affect other processes involved in photosynthesis. For instance, the imposition of drought leads to alterations in the plant water relations, stomatal closure, reduced biochemical activities, and decreased efficiency in energy conversion processes (Bellasio et al. 2023). These changes can impact intercellular and intracellular CO_2_ and bicarbonate diffusion, light interception, structural integrity, and nutrient uptake, all of which have a depressive effect on assimilation (Lawlor and Cornic 2002). When a population of experimental datapoints of *A* are plotted over *g*_SC_, the response of *A* will therefore aggregate both the impact of *g*_S_ on *A* and the influence of other drivers on *A*, hence include both stomatal and non-stomatal effects. As a result, the slope of a regression fitted through experimental data points of *A* against *g*_SC_ is typically not indicative of $\partial A/\partial {g}_{\mathrm{SC}}$. As Wong (1979) wrote, “linear relationships between *A* and *g*_S_ do not necessarily indicate that stomata control the rate of assimilation”. Postulating a link between stomatal control and the slope of the regression fitted to a set of empirical *A* and *g*_SC_ data pairs becomes particularly problematic when comparing C_3_ and C_4_ plants. This is due to the fact that regression lines of* A* to *g*_SC_ are typically steeper in C_4_ plants than in C_3_ plants (Quirk et al. 2019); however, C_4_ plants usually have less advantage than C_3_ plants when they open their stomata (Farquhar and Sharkey 1982). I will now illustrate this numerically.

**Fig. 1:**
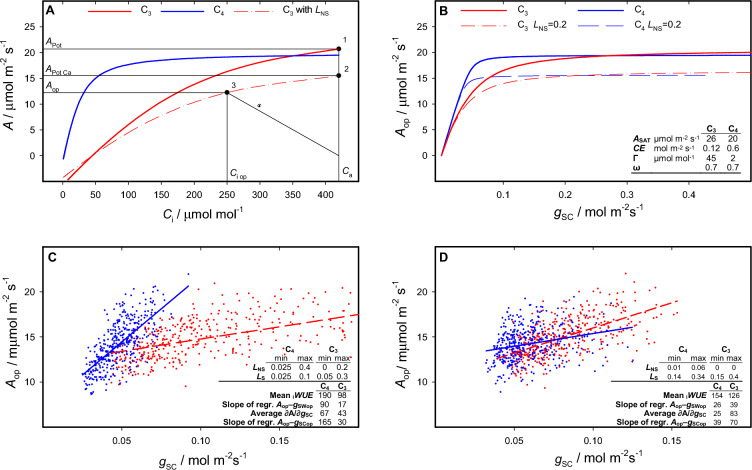
Simulation of _i_WUE, $\partial A/\partial {g}_{\mathrm{SC}}$, and regression of *A* on *g*_S_. Panel **A** shows the modelled dependence of C_3_ (thick dashed red line) and C_4_ (thick solid blue line) assimilation (*A*) upon CO_2_ concentration in the substomatal cavity (*C*_i_, Eq. 1), parameterised to generically represent the photosynthetic optimum for young leaves under moderate light intensity (inset in Panel **B**). The rationale of the simulations is exemplified for *C*_3_ assimilation. Point 1 (*C*_a_,* A*_Pot_), lying on the uppermost *A*/*C*_i_ curve, shows the potential assimilation attained if intercellular spaces were directly exposed to external CO_2_ concentration, *C*_a_ (Eq. 1 calculated for *C*_i_ = *C*_a_). In Point 2 (*C*_a_,* A*_PotCa_) assimilation is only limited by non-stomatal limitations (*L*_NS_, Eq. 2); the thin dashed line is the *C*_3_ *A*/*C*_i_ curve passing through *A*_PotCa_, derived by reducing saturated rate (*A*_SAT_) and initial slope (CE) proportionally (Eq. 3). Point 3 (*C*_iop_,* A*_op_) is a generic operational point where stomatal limitation (*L*_S_) may occur (Eq. 4); the slope of the supply function ***s*** (Eq. 6) is *g*_SC_. Panel **B** shows the response of assimilation to *g*_SC_ obtained under a *C*_a_ of 420 μmol mol^−1^ for plants operating at their optimum, or with non-stomatal limitation (*L*_NS_ = 0.2). The inset shows model inputs, modified from Bellasio et al. (2016a, b) to generically describe C_3_ and C_4_ assimilation. Panel **C** shows the plot of *A*_op_ and *g*_SCop_ obtained using 400 random combinations of *L*_NS_ and *L*_S_ between a minimum and maximum shown in the inset. Outputs in the inset are the mean _i_WUE (*A*_op_/*g*_SWop_) calculated for 400 (*g*_SWop_,* A*_op_) points; the mean of the $\partial A/\partial {g}_{\mathrm{SC}}$ of the *A*/*g*_SC_ curves passing through each of the 400 (*g*_SCop_,* A*_op_) points, and the slope of the regression fitted to the same points after adding normally distributed noise with a standard deviation of 10% of the simulated *A*_op_ and *g*_S_. Panel **D** shows the plot of *A*_op_ and *g*_SCop_ obtained using the* L*_NS_ and *L*_S_ limits shown in the inset, output as in Panel **C**. Units of slopes and _i_WUE are $\frac{\mu \,\mathrm{mol }\,{\mathrm{CO}}_{2}}{\mathrm{mol Air}}$

To calculate operational values of *A* and *g*_S_ starting from input values of stomatal and non–stomatal limitations (*L*_S_ and *L*_NS_), I start from describing the dependence of *A* upon *C*_i_ with a function after Prioul and Chartier (1977) in the formulation of Bellasio et al. (2016b) as:

1 $${A}=\frac{{\text{CE}} \left({C}_{\mathrm{i}}-\varGamma \right) + {A}_{\mathrm{SAT}}-\sqrt{{({\text{CE}}[{C}_{\mathrm{i}}-\varGamma ]+{A}_{\mathrm{SAT}})}^{2}-(4 \omega {A}_{\mathrm{SAT}} {\text{CE}} [{C}_{\mathrm{i}}-\Gamma ]})}{2\omega },$$

where *A*_SAT_ is the CO_2_-saturated rate, CE is the initial slope, *Γ* is the *x*-intercept, *ω* is defining curvature. Typical *A*/*C*_i_ curves (Fig. 1A) were obtained using a generic C_3_ and C_4_ parameterisation (inset in Fig. 1B).

The potential assimilation that would occur if intercellular spaces were directly exposed to *C*_a_ is calculated by substituting *C*_a_ for *C*_i_ in Eq. 1 (*A*_Pot_ Fig. 1A, point 1). Ordinarily, non-stomatal limitation (*L*_NS_) may reduce assimilation to a point (Fig. 1A, point 2) called *A*_PotCa_, which, from the definition of non-stomatal limitation (Bellasio et al. 2018) is:

2 $${A}_{\mathrm{PotCa}}={A}_{\mathrm{Pot }}\left(1-{L}_{\mathrm{NS}}\right).$$

To find the *A*/*C*_i_ curve passing through *A*_PotCa_ one can assume that CE and *A*_SAT_ scale linearly to the values of CEʹ and *A*_SAT_ʹ. If the ratio $\mu ={A}_{\mathrm{SAT}}/{\text{CE}}$ is invariant, then the standard quadratic form of Eq. 1, $\left\{\omega {A}^{2}- \left({\text{CE}}\left[{C}_{\mathrm{i}}-\Gamma \right]+{A}_{\mathrm{SAT}}\right)A+ {A}_{\mathrm{SAT}} {\text{CE}} \left[{C}_{\mathrm{i}}-\Gamma \right]=0\right\},$ can be solved for CEʹ as:

3 $${\text{CE}}^{\prime} = \frac{{A(\mu + C - \Gamma ) + \sqrt {[A(\Gamma - C - \mu )]^{2} - [4\omega A^{2} \mu (C - \Gamma )]} }}{{2\mu (C - \Gamma )}}$$

CEʹ, the estimated value of CE of the leaf in the presence of *L*_NS_, is calculated by inputting *A*_PotCa_ (Eq. 2) and *C*_a_ in Eq. 3;* A*_SAT_ʹ, the estimated value of *A*_SAT_ in the presence of *L*_NS_, is, by assumption, *μ* CEʹ.

Stomatal limitation (*L*_S_) would further reduce assimilation to the ‘operational’ *A*_op_ (Fig. 1A point 3), which, from the definition of stomatal limitation (Farquhar and Sharkey 1982) is:

4 $${A}_{\mathrm{op}}={A}_{\mathrm{PotCa}}-{A}_{\mathrm{Pot}} {L}_{\mathrm{S}}.$$

The new corresponding *C*_iop_ is found by solving Eq. 1 as:

5 $$C_{\text{iop}} = \frac{{\omega A_{\text{op}} ^{2} + {\text{CE}}^{\prime}\Gamma A_{\text{op}} - A_{\text{SAT}} ^{{\prime 2}} A_{\text{op}} - A_{\text{SAT}} ^{\prime } {\text{CE}}^{\prime}\Gamma }}{{{\text{CE}}^{\prime}A_{{{\text{op}}}} - A_{\text{SAT}} ^{\prime } {\text{CE}}^{\prime}}}.$$

Finally, *g*_SC_ is:

6 $${g}_{\mathrm{SC}}=\frac{{A}_{\mathrm{op}}}{{C}_{\mathrm{a}}-{C}_{\mathrm{iop}}}.$$

The general behaviour of the model is shown if Fig. 1B. When there is only *L*_S_, at any given *g*_SC_ the points (*C*_iop_,* A*_op_) satisfying Eq. 1 lie on the upper *A*/*C*_i_ curves. Introducing *L*_NS_ lowers *A*_op_ mainly when *g*_SC_ is high.

Four hundred random combinations of *L*_NS_ and *L*_S_ were generated in a manner that they fall within the minimum and the maximum intervals specified in the inset of Fig. 1C, and their sum would be within the range of 0.05 to 0.5. The proportion of *L*_S_ was intentionally set lower for C_4_ assimilation than for C_3_, replicating physiological operational conditions (Ghannoum et al. 2003; Bellasio et al. 2018). Equations 4 and 6 were employed to simulate *A* and *g*_SC_, respectively, 10% normally distributed-error was added, and a regression line was fitted to the resulting (*C*_iop_,* A*_op_) pairs (Fig. 1C). Each (*C*_iop_,* A*_op_) pair corresponds to an *A*/*C*_i_ curve (Eq. 1) parameterised with CEʹ, *A*_SAT_ʹ (Eq. 3), *ω* and *Γ* (inset in Fig. 1B). For each of these *A*/*C*_i_ curves, $\partial A/\partial {g}_{\mathrm{SC}}$ was calculated numerically at each (*C*_iop_,* A*_op_) point for small increments of *L*_S_ in Eq. 3.

Despite imposing a physiologically lower proportion of *L*_S_ in C_4_ assimilation, the slope of the regression line fitted to the simulated data points was higher for C_4_ than C_3_ assimilation. The average $\partial A/\partial {g}_{\mathrm{SC}}$ of the individual *A*/*g*_SC_ curves passing through the (*g*_SCop_,* A*_op_) pairs differed from the slope of the regression line fitted through the same (*g*_SCop_,* A*_op_) pairs (insets of Fig. 1C and 1D). This means that the slope of the regression line fitted through operational *A*_op_ and *C*_iop_ does not serve as a measure of *L*_S_ or of $\partial A/\partial {g}_{\mathrm{SC}}$.

## The slope of the regression of *A* on *g*_SW_ is not water use efficiency

The slope of the regression for a set of gas exchange points measured in operational conditions (*g*_SWop_,* A*_op_) has been erroneously employed to measure the average _i_WUE of a set of data points, as seen in studies like Vogan and Sage (2011) and Killi et al. (2017). However, the slope of a regression fitted to a set of (*g*_SWop_,* A*_op_) pairs would equal their average _i_WUE only if the unconstrained regression line passed through the origin, which is typically not the case.

To illustrate this numerically, four hundred random combinations of *L*_NS_ and *L*_S_ were generated in a way that they fall within the minimum and the maximum intervals specified in the inset of Fig. 1D. Subsequently, assimilation and *g*_SC_ were simulated as described above, and a regression line was fitted to the resulting (*g*_SCop_,* A*_op_) pairs (Fig. 1D) and (*g*_SWop_,* A*_op_) pairs. The slope of the regression fitted through the (*g*_SWop_,* A*_op_) pairs (inset in Fig. 1D) was lower in C_4_ than in C_3_, but the ranking of _i_WUE was in the opposite order (inset in Fig. 1D). This shows that the slope of the regression of *A*_op_ on *g*_SW_ may not even scale to _i_WUE, and therefore should not be employed as a measure of _i_WUE.

Instead of by fitting a regression line through the (*g*_SWop_,* A*_op_) pairs, water use efficiency should be calculated for each individual data point as ${}_{\mathrm{i}}{\text{WUE}}=A/{g}_{\mathrm{SW}}$. The implementation is entirely straightforward, because gas exchange measurements automatically provide the necessary information (*A* and *g*_SW_).

## Conclusion

I have provided numerical evidence that inferring water use efficiency or stomatal control over assimilation from the slope of the empirical regression of operational CO_2_ assimilation on stomatal conductance is inappropriate. I recommend calculating both metrics: ‘intrinsic water use efficiency’ (representing the assimilation-to-stomatal conductance ratio that is independent of air humidity and therefore preferable for plant comparisons) and ‘instantaneous transpiration efficiency’ (the term I suggest reserving for the assimilation-to-transpiration ratio), for each individual gas exchange data point.

## Data Availability

Code and output available upon request.
